# Analysis of large versus small dogs reveals three genes on the canine X chromosome associated with body weight, muscling and back fat thickness

**DOI:** 10.1371/journal.pgen.1006661

**Published:** 2017-03-03

**Authors:** Jocelyn Plassais, Maud Rimbault, Falina J. Williams, Brian W. Davis, Jeffrey J. Schoenebeck, Elaine A. Ostrander

**Affiliations:** Cancer Genetics Branch, National Human Genome Research Institute, National Institutes of Health, Bethesda, Maryland, United States of America; Clemson University, UNITED STATES

## Abstract

Domestic dog breeds display significant diversity in both body mass and skeletal size, resulting from intensive selective pressure during the formation and maintenance of modern breeds. While previous studies focused on the identification of alleles that contribute to small skeletal size, little is known about the underlying genetics controlling large size. We first performed a genome-wide association study (GWAS) using the Illumina Canine HD 170,000 single nucleotide polymorphism (SNP) array which compared 165 large-breed dogs from 19 breeds (defined as having a Standard Breed Weight (SBW) >41 kg [90 lb]) to 690 dogs from 69 small breeds (SBW ≤41 kg). We identified two loci on the canine X chromosome that were strongly associated with large body size at 82–84 megabases (Mb) and 101–104 Mb. Analyses of whole genome sequencing (WGS) data from 163 dogs revealed two indels in the *Insulin Receptor Substrate 4* (*IRS4*) gene at 82.2 Mb and two additional mutations, one SNP and one deletion of a single codon, in *Immunoglobulin Superfamily member 1* gene (*IGSF1*) at 102.3 Mb. *IRS4* and *IGSF1* are members of the GH/IGF1 and thyroid pathways whose roles include determination of body size. We also found one highly associated SNP in the 5’UTR of *Acyl-CoA Synthetase Long-chain family member 4* (*ACSL4)* at 82.9 Mb, a gene which controls the traits of muscling and back fat thickness. We show by analysis of sequencing data from 26 wolves and 959 dogs representing 102 domestic dog breeds that skeletal size and body mass in large dog breeds are strongly associated with variants within *IRS4*, *ACSL4* and *IGSF1*.

## Author summary

Modern dog breeds display significant variation in body size and mass resulting from selective breeding practices. A genome-wide association study (GWAS) of 170,000 SNPs genotyped on a panel of 855 dogs from 88 breeds revealed two loci on the canine X chromosome that were strongly associated with the large Standard Breed Weight (SBW >41kg [90lb]) in domestic dog breeds. Fine mapping of both loci using whole genome sequencing (WGS) data from 163 dogs highlighted three candidate genes, *IRS4* and *ACSL4* within the first locus (82–84 Mb), and *IGSF1* at the second locus (101–104 Mb). Associated variants were found in all three genes. Of interest, the *IRS4* gene is involved in the IGF-1/growth hormone pathway and *IGSF1* is associated with human obesity. *ACSL4* is associated with muscling and back fat thickness in pigs, a phenotype we observe in “bulky” dog breeds. This work identifies three new genes and associated variants that contribute to body mass in dogs, advancing our understanding of morphologic variability in domestic dog breeds.

## Introduction

Body size variation observed across domestic dog (*Canis lupus familiaris*) breeds provides one of the most visual examples of human selection. Dogs are thought to have been domesticated about 15,000–30,000 years ago [1–10], with the grey wolf being the closest living ancestor [2,4,11–14]. The majority of modern dog breeds, however, were developed within the past 300 years with over 340 official breeds noted worldwide [15,16].

The creation of breeds requires codified standards that describe the physical characteristics of the dog. The breeding strategies used to create dogs with highly specific features have resulted in relatively isolated, pure breeding populations [17]. The same selective pressures that have reduced phenotypic and genotypic heterogeneity within breeds [6,10,18–21] result in long stretches of linkage disequilibrium (LD) in dogs [20,22–24]. Given these advantageous features, studies of dog breeds have led to the identification of disease genes of interest for human health and biology, including rare human disorders [25–28], *e*.*g*. cancer [29–33]. The same genomic characteristics have also produced a stellar system for identifying the genes underlying both simple and complex morphologic traits, including coat color and texture variation, tail curl, ear position, skull shape, chondrodysplasia and body size (reviewed in [34–39]).

Body size is the most striking of these traits, as the difference in skeletal size from the smallest to largest dog breeds is about 40-fold [15,16]. Initial studies of dog body size focused on the Portuguese Water Dog (PWD), a breed for which the American Kennel Club (AKC) permits about a 50% level of size variation amongst members of the breed [40]. A genome-wide association study (GWAS) of PWD representing a range of body sizes identified the *insulin-like growth factor-1 gene* (*IGF1*) [41], and additional studies of Miniature Poodles and Dachshunds implicated the *IGF1 receptor* (*IGF1R*) as well [42], both of which are important regulators of body size. The *IGF1* pathway has also been established as important in normal stature in humans, and mutations in *IGF1* have been shown to reduce body size in mice [43–46].

Four additional positional candidate genes contributing to variation in canine body size have been identified: the *Growth Hormone Receptor* (*GHR*) on canine chromosome 4 (CFA4); *High Mobility Group AT-hook 2* (*HMGA2*) gene on CFA10; *Stanniocalcin 2* (*STC2)* on CFA4; and *SMAD family member 2 (SMAD2)* on CFA7 [22,47,48]. The most closely associated variants have been reported for each [47]. This includes two non-synonymous SNPs in exon 5 of *GHR*, a SNP in the 5’UTR of *HMGA2*, a SNP located 20 kb downstream from *STC2* and a 9.9 kb deletion 24 kb downstream from *SMAD2*, all of which are highly associated with lower Standard Breed Weights (SBW) [47]. Additional studies noted ten additional putative loci [23,48]. These studies did not, however, identify causal variants and did not ascertain the contribution of each gene, or a combination of genes, to overall size variance in dogs.

Variant haplotypes of the six genes described above are strongly associated with large versus small body size, although some exceptions exist. While we showed previously that *IFG1*, *IFG1R*, *SMAD2*, *HMGA2*, *STC*, and *GHR* variants account for about 60% of body size variance in breeds with a SBW ≤41 kg (90 lb) which are referred hereto as “small/medium breeds,” the same genes account for <5% of variance in breeds with a SBW >41 kg, hereto referred to as “large breeds”. We initially identified two loci on the X chromosome spanning several megabases (Mb) as contributors to body size in large breeds through a GWAS of 915 dogs representing 80 domestic dog breeds [22]. The result has since been replicated by several groups [22,23,47,48]. No study, however has explored the result in detail, in part because the lack of heterozygosity on the canine X chromosome can reflect popular sire effects, which may complicate fine mapping efforts.

In this study we investigate body size loci on the X chromosome using SNP chip data from ≥800 dogs [21], together with whole genome sequencing (WGS) data. We show that both of the previously identified loci are strongly associated with large breeds, and we perform fine mapping at each locus using WGS data from 163 breeds. Together, these data reveal associations with three excellent positional candidate genes: *Insulin Receptor Substrate 4* (*IRS4*) which interacts with multiple growth factor receptors such as *IGF1R* [49], *Immunoglobulin superfamily member 1* (*IGSF1)* which is involved in the biosynthesis of thyroid hormones [50–52] and *Acyl-CoA Synthetase Long-chain family member 4* (*ACSL4*) which plays a role in lipid biosynthesis and fatty acid degradation [53].

## Results

### Genome-wide association study

We initially genotyped a large dataset of 855 dogs representing 88 breeds on the Illumina 170k Canine HD Array [21]. For purposes of this analysis, large breeds included 165 dogs from the 19 following giant breeds: Akita, Anatolian Shepherd Dog, Bernese Mountain Dog, Black Russian Terrier, Bullmastiff, Dogue de Bordeaux, English Mastiff, Great Dane, Greater Swiss Mountain Dog, Great Pyrenees, Irish Wolfhound, Kuvasz, Leonberger, Neapolitan Mastiff, Newfoundland, Rottweiler, Saint Bernard, Scottish Deerhound, and Tibetan Mastiff. Using the array data, we compared the genotypes from the above large breeds to 690 dogs from 69 small/medium breeds (S1 Table). To correct for cryptic relatedness and sex, we used GEMMA [54,55], a linear mixed-model method which accounts for population stratification and relatedness.

**Fig 1:**
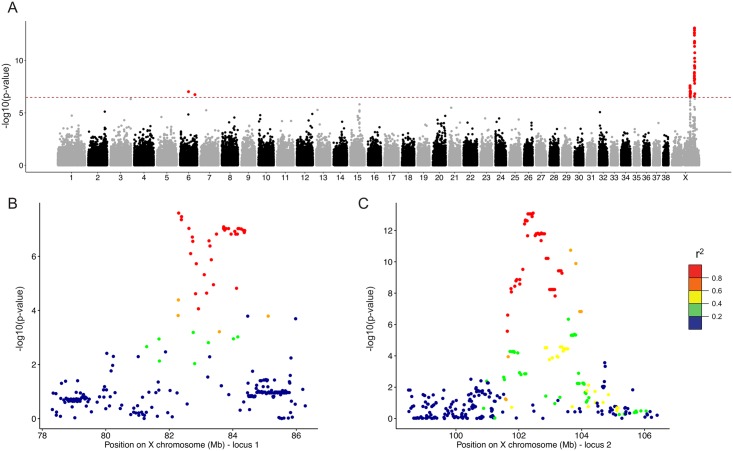
GWAS results for 165 large breed dogs *vs*. 690 medium/small breed dogs. (A) Manhattan plot for the GWAS is shown. The -log_10_ P-values for each SNP are plotted on the y-axis *versus* each canine autosome and the X chromosome on the x-axis. The red line represents the Bonferroni corrected significance threshold (-log_10_ (P) = 6.48) and SNPs passing this threshold are colored in red. (B and C) Regional plot for genome-wide significant association on the X chromosome for 78–86 Mb (B) and 98–106 Mb (C). Each plot spans the genomic regions from 4000 kb upstream to 4000 kb downstream of the most significant SNP at each locus. SNPs are colored based on the strength of LD values (r^2^ values) considering the most strongly associated SNP and the other SNPs in the region.

A total of 81 SNPs were significant at a genome-wide level for the trait of body mass, and which passed the Bonferroni significance threshold (-log_10_(P) >6.48) (Fig 1A). Among these, we identified two primary loci on the X chromosome (Table 1). The first locus (locus 1) included 23 SNPs, and spanned 82,296,039 to 84,376,308 bp (Fig 1B). A stronger signal (P = 7.74x10^-14^) was identified at a second locus on the X chromosome, which spans 101,646,292 to 103,984,352 bp, corresponding to 56 additional SNPs that passed the significance threshold (Fig 1C). Neither of these loci were within the pseudoautosomal region of the X. Two additional SNPs located on CFA6 passed the significance threshold, chr6: 38,284,916 (P = 9.36x10^-8^) and chr6: 67,350,922 (P = 1.80x10^-7^), but no additional associated SNPs were found in these regions and the result was not explored further at this time.

**Table 1:** Best SNP markers associated with SBW above 41 kg in dogs.

Rank	Chromosome	Position	Major Allele	Minor Allele	Allele Frequency	P-Wald	Closest Gene
1	X	102455197	G	A	0.425	7.74E-14	Olfactory Receptor Cluster
2	X	102292529	A	G	0.42	8.75E-14	Olfactory Receptor Cluster
3	X	102323911	A	G	0.42	8.75E-14	*IGSF1*
4	X	102333672	A	G	0.42	8.75E-14	*IGSF1*
5	X	102360497	C	A	0.42	8.75E-14	*IGSF1*
6	X	102381223	G	A	0.42	8.75E-14	*IGSF1*
7	X	102385409	G	A	0.42	8.75E-14	*IGSF1*
8	X	102415621	C	A	0.42	8.75E-14	Olfactory Receptor Cluster
9	X	102429959	A	C	0.42	8.75E-14	Olfactory Receptor Cluster
10	X	102423378	A	T	0.42	1.29E-13	Olfactory Receptor Cluster
…	…	…	…	…	…	…	…
54	X	82296039	A	G	0.391	2.51E-08	*IRS4*
55	X	82387385	A	C	0.395	3.41E-08	*IRS4*
56	X	82381884	G	A	0.408	4.41E-08	*IRS4*
57	X	83713308	A	G	0.386	8.10E-08	x
58	X	82619550	A	G	0.395	9.27E-08	*GUCY2F*
59	6	38284916	G	A	0.022	9.36E-08	*SRRM2*

We examined the loci of interest more closely by calculating pairwise linkage disequilibrium (LD) between SNPs within the 4,000 kilobase (kb) regions surrounding the most strongly associated SNPs at each of the two loci on the X chromosome. Thirty-five SNPs were highly correlated (pairwise r^2^ >0.8) at locus 1 (Fig 1B) while 53 were highly correlated at locus 2 (Fig 1C). We next investigated each locus by focusing on regions in which SNPs had pairwise r^2^ values >0.5 and extending these regions by +/- 200 kb. The two refined intervals ranged from 82,079,576 to 84,576,308 for locus 1 and from 101,378,080 to 104,418,823 for locus 2. Locus 1 contains 17 annotated protein-coding genes and 11 annotated RNA genes (small RNAs and long non-coding RNAs) or pseudogenes (S1 Fig). Among these genes, the strongest candidate gene to emerge at locus 1 is *Insulin Receptor Substrate 4* (*IRS4)*, a gene involved in the thyroid hormone pathway, which is associated with IGF1R signaling and body mass index [49,56]. At locus 2, 20 protein-coding genes are annotated, including a cluster of olfactory receptor genes, and seven noncoding RNAs including microRNA, noncoding RNA and pseudogenes (S2 Fig). From these 20 genes, we identified one striking candidate, *Immunoglobulin Superfamily member 1 (IGSF1*), that encodes an immunoglobulin in the thyroid hormone pathway, and which was previously associated with obesity in IGSF1-deficient humans [50–52].

### Fine mapping strategy

To identify functional variants within these two critical intervals, notably in the two strongest candidate genes, *IRS4* and *IGSF1*, we used WGS data from 163 purebred dogs inclusive of 87 breeds representing the full range of body height and weight specified by the American Kennel Club (AKC). Each WGS had a mean read depth of at least 10x (S2 Table). Among these, 21 dogs from 14 breeds were considered large (SBW >41 kg [90 lb]), including the English Mastiff, Irish Wolfhound and Saint Bernard. We first filtered to retain biallelic variants including SNPs and small insertions or deletions <100 bp, with a minor allele frequency (MAF) >0.05. We next screened the remaining biallelic variants, keeping only variants for which the major allele frequency in large breeds was >0.5 and <0.5 in small/medium breeds (SBW ≤41 kg [90 lb]). A total of 6,809 variants remained for locus 1 and 1,997 variants for locus 2 (S3 and S4 Tables). Using these biallelic variant datasets, we performed a new association study for both loci using GEMMA, a linear mixed-model software [54,55], thus defining which alleles were the most strongly associated with large breeds, and in each case that allele was termed the “large allele”.

### Fine mapping results at locus 1

The 6,809 variants identified at locus 1 define a set of genotypes which correspond to a single large haplotype present in more than 90% of large breeds (S1 Fig). This spans the strong signal originally identified in the GWAS presented here. Among these variants, we identified one codon deletion (chrX.g.82288614-82288616delTCG) and one insertion (chrX.g.82288998-82288999insGCT) both in the exonic region of the *IRS4* gene that were in LD with one another (S1 Fig). Neither, however, are likely to be significant for this study as neither mutation changes the IRS4 protein size, distinguishes between various size breeds or is in a well-conserved region (Table 2). In addition, for each variant the “large alleles” were also identified in more than 20% of small/medium breeds.

**Table 2:** Conservation of *IRS4* mutated codons (in bold) between mammals.

Species	IRS4 Protein Size	% Identity with dog	Codon Insertion (codon 1126)	Codon Deletion (codon 1253)
Large Dog (SBW >41 kg [90 lb])	1278	-	LERDLPASLASAA**A**AAAASAAAPALAL	PPEQQVSDNDDDDDD**-**DTYVRMDF
Medium & Small Dog (SBW ≤41 kg)	1278	-	LERDLPASLASAA-AAAASAAAPALAL	PPEQQVSDNDDDDDD**D**DTYVRMDF
Human	1257	82.6	LERDLSPS----SAPAVASAAEPTLAL	PPEREDSDNDDD-----THVRMDF
Mouse	1216	74.2	LDRDFPA----ASAVIAAPAEAPLLAV	RPQERADSEDDDDDD**D**DIYVRMDF
Cat	1124	86.0	LERDLSASL--------ASVAAPALAV	PRER-----EDSDDD**D**DTYVRMDF
Cow	1255	81.5	LERDLSASFAAAAAAV------PTLAL	PPERE------DSDD**D**DTYVRMDF
Horse	1051	82.4	LERDLSASLASAAALAA----APTLGV	PPEQE-----DSDDD**D**DTYVRMDF
Megabat	1260	76.3	LERDLYASFASAAVAVVV----PTFAL	LPERE-----DSDDD**E**DTYARMDF

While we discarded the above variants in *IRS4* from an association with body size, a re-analysis aimed at finding structural variants revealed a large 56 kb deletion (ChrX:82455513–82511744) located 150 kb upstream from the starting codon of *IRS4* (S1 Fig). The variant was only present in the Bernese Mountain Dog, Black Russian Terrier, English Mastiff, Greater Swiss Mountain Dog, Rottweiler, and Saint Bernard. Visualization of the deletion on an agarose gel indicated that it was also present in multiple other large breeds: the Alaskan Malamute, Bouvier des Flandres, Bullmastiff, Dogo/Presa Canario, Dogue de Bordeaux, Kuvasz and Leonberger.

### *ACSL4* gene and the large muscled phenotype at locus 1

**Fig 2:**
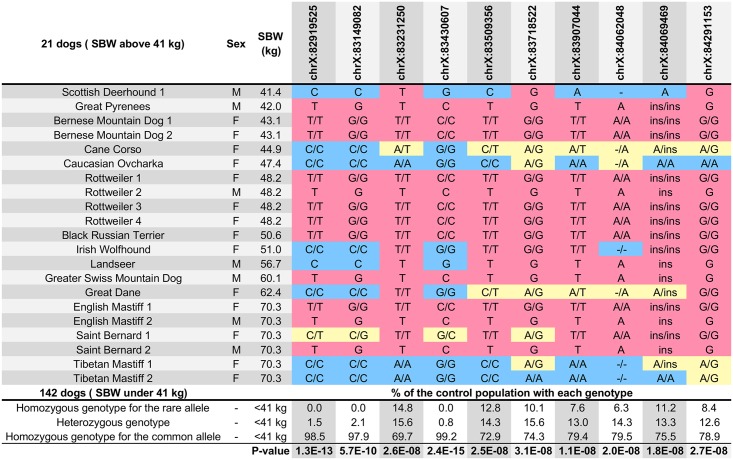
Observed genotypes for the ten most strongly associated variants identified in large dogs using WGS data. The three first columns correspond to the dog, breed, sex and standard breed weight (SBW). The next 10 columns correspond to the 10 most strongly associated variants at locus 1, identified from WGS data. The first part of the table corresponds to large dogs (SBW >41 kg). Homozygous and hemizygous genotypes for the “large allele” are colored in red, homozygous/hemizygous genotypes for the “small/medium” allele are colored in blue and heterozygous genotypes are colored in yellow. The second part of the table shows the distribution of the “large allele” in the 140 dogs with a SBW ≤41 kg. Values correspond to the percentage of this control population showing each genotype by variant. The last row shows the respective p-value estimated (Wald test) for each variant.

Among the 6,809 biallelic variants identified at locus 1, we also found three variants, distinct from the above, which were themselves in LD (Fig 2), and which harbored the highest p-values (10^−10^<P-value<10^−15^, P-Wald test) (Table 3). One of the three is a SNP (chrX.g.82919525G>A) in the 5’UTR of *Acyl-CoA Synthetase Long-chain family member 4* (*ACSL4*), a gene which plays a role in lipid biosynthesis and fatty acid degradation [53]. This nucleotide is included in a highly conserved region also identified in the human and mouse genomes (S3 Fig). The other two SNPs were intergenic or intronic (in *AMMECR1*) (Table 3).

**Table 3:** Best variants associated with SBW above 41 kg in dogs, identified on the locus 1 (CFAX) using 163 WGS data.

Position	Major Allele	Minor Allele (Large Allele)	Allele Frequency	Non-missing Allele Count	P-Wald	Closest Gene	Details
83430607	G	C	0.08368	239	2E-15	*AMMECR1*	Intronic SNP
82919525	C	T	0.08475	236	1E-13	*ACSL4*	5'UTR SNP
83149082	C	G	0.09574	188	6E-10	*SH3D21*	7.6 kb upstream of the start codon
83907044	A	T	0.2426	235	1E-08	*CHRDL1*	Intronic SNP
84069469	A	ATAATAATAATAATAATAG	0.2947	190	2E-08	*PAK3*	4.9 kb upstream of the start codon
84062048	-	A	0.2392	209	2E-08	*PAK3*	13 kb upstream of the start codon
83509356	C	T	0.2833	240	2E-08	*PAK3*	13 kb upstream of the start codon
83232150	A	T	0.3259	224	3E-08	*TMEM164*	Intronic SNP
84291153	A	G	0.2655	177	3E-08	*PAK3*	Intronic SNP
83718522	A	G	0.2913	206	3E-08	*SNU13*	Intergenic

All three variants, the SNP in *ACSL4*, together with the two SNPs in the same LD block, were present in an interesting subset of large dogs. Specifically, variants were only identified in four of 19 large breeds: Bernese Mountain Dog, Greater Swiss Mountain Dog, Rottweiler, and Saint Bernard. The other 78 breeds, which included large, medium and small breeds, lacked all three variants. Of note, all three variants were missing in several large breeds that were skeletally quite large, but comparatively lean, including the Cane Corso, Great Dane, and Irish Wolfhound, among others (Fig 2). The breeds in which the variant is found are not simply skeletally large, but also considered “bulky,” with considerable muscle and fat.

**Fig 3:**
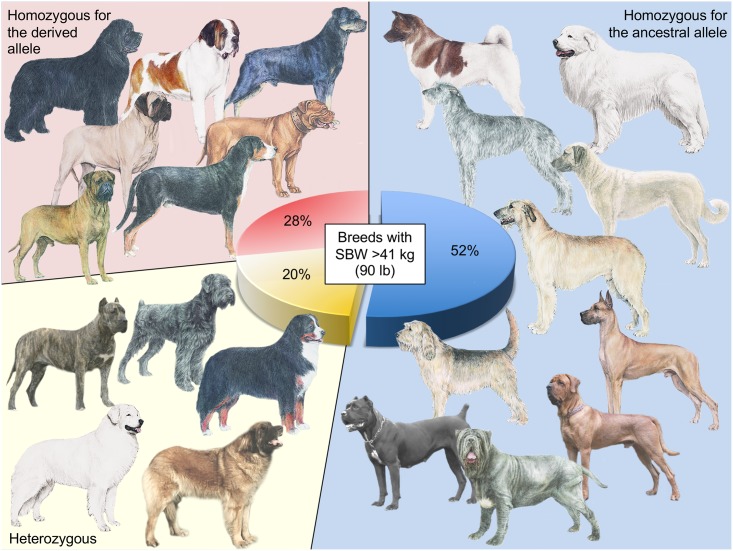
Distribution of the derived *ACSL4* allele in the large dog breeds. The Bullmastiff, Dogue de Bordeaux, English Mastiff, Greater Swiss Mountain Dog, Newfoundland, Rottweiler and the Saint Bernard are homozygous for the derived allele (red) while Bernese Mountain Dog, Black Russian Terrier, Dogo/Presa Canario, Kuvasz and Leonberger were either homozygous or heterozygous (yellow) at *ACSL4*. Other large dog breeds (*e*.*g*. Akita, Anatolian Shepherd Dog, Cane Corso, Great Dane, Great Pyrenees, Irish Wolfhound, Neapolitan Mastiff, Otterhound, Scottish Deerhound, Tibetan Mastiff, Tosa Inu), wild canids and all medium/small breeds carry the ancestral allele in the homozygous state (blue). Pictures provide by the American Kennel Club (AKC) and Larousse.

We next checked for the frequency of the 5’UTR *ACSL4* variant by testing a larger panel of 959 dogs from 102 breeds, which represented an additional 54 breeds (S5 Table). The “bulky allele” was present in several dogs with a bulky, heavily muscled body: Bullmastiff, Dogue de Bordeaux, English Mastiff, Greater Swiss Mountain Dog, Newfoundland, Rottweiler and Saint Bernard, where it appears fixed in nearly 100% of dogs from each breed (Fig 3). We found that the Alaskan Malamute, Bernese Mountain Dog, Black Russian Terrier, Bouvier des Flandres, Dogo/Presa Canario, Kuvasz and Leonberger breeds could be either heterozygous or homozygous for both alleles. In total, 48% of the large breeds shared the “bulky allele” (heterozygous or homozygous) (Fig 3). Sanger sequencing of a larger panel of dogs (≥10 dogs per breed) including the Anatolian Shepherd Dog, Great Dane, Great Pyrenees, Irish Wolfhound, Neapolitan Mastiff, and Scottish Deerhound confirmed the absence of the “bulky allele” in these breeds, many of which are long and lean rather than bulky. Of note, the *ACSL4* variant mutation was never observed in medium or small breeds, even small muscled breeds such as American Staffordshire Terrier, Boston Terrier, or Bulldog. The results were the same with the two intergenic or intronic variants in LD with the 5’ UTR *ACSL4* variant (S5 Table).

**Fig 4:**
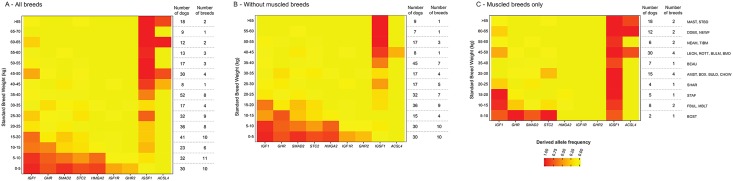
Derived allele frequencies at multiple loci involved in body weight and size variations in (A) modern dog breeds, (B) modern dog breeds without inclusion of muscled breeds, (C) only muscled breeds. The frequency of the derived allele in 5-kg weigh classes is represented on a color scale. Dogs with a SBW above 65 kg are collapsed in a single category (>65 kg) due to the lack of genotype variation in the group at these markers. Muscled breeds include the Boston Terrier (BOST), French Bulldog (FBUL), Miniature Bull Terrier (MBLT), Staffordshire Bull Terrier (STAF), Chinese Shar-pei (SHAR), Bulldog (BULD), Chow-Chow (CHOW), American Staffordshire Terrier (AMST), Boxer (BOX), Beauceron (BEAU), Bullmastiff (BULM), Bernese Mountain Dog (BMD), Rottweiler (ROTT), Leonberger (LEON), Tibetan Mastiff (TIBM), Neapolitan Mastiff (NEAM), Newfoundland (NEWF), Dogue de Bordeaux DDBX), English Mastiff (MAST) and Saint Bernard (STBD).

Sanger sequencing of the set of wild canids (24 grey wolves, two red wolves and two coyotes) confirmed that the three mutations, including the *ACSL4* variant, are absent from the wild canid population, leading us to consider these variants as derived alleles which were likely selected by humans to create large and muscled breeds. The *ACSL4* gene is associated with the traits of heavy muscling and “back fat thickness” in pigs, a phenotype that aptly describes the breeds carrying the mutation [57–61]. We conclude that *ACSL4*, potentially in concert with the upstream deletion in *IRS4*, is needed to create the large bulky/muscled phenotype observed in the breeds reported here (Figs 3 and 4).

### Fine-mapping using Whole-Genome Sequencing (WGS) data at locus 2

The 1,997 variants at locus 2 also define a large homozygous haplotype found in all large breeds, except the Scottish Deerhound (S2 Fig). The haplotype found in the large breeds is also observed in 24 of 66 small/medium breeds including the Boston Terrier, Boxer, French Bulldog, Irish Water Spaniel and Labrador Retriever. Not unexpectedly, we observe the heterozygous state in 19 additional small breeds. While WGS demonstrates that the haplotype is found in breeds of varying size, the fact that it is present in 18 of 19 breeds in the homozygous state suggest that it is necessary, but not sufficient, for large body mass. Within this region, we detected missense changes in three genes: *ARHGAP36* (*Rho GTPase Activating protein 36*), *IGSF1* (*Immunoglobulin Superfamily Member 1*) and *FRMD7* (*FERM Domain Containing 7*) (S4 Table).

Since the *IGSF1* gene is a strong candidate for body size [50–52], we examined it further, noting three variants in the canine sequence (S2 Fig). The first is a single nucleotide change in the 3’UTR (chrX.g.102360204G>A; rs24856221), but the distribution of the genotype in the dog population suggests that it was not associated with SBW (S4 Table). The second is a missense mutation in exon 12 (chrX.g.102364864T>G; rs852386368) that changes an aspartic acid to a glutamic acid (ENSCAFP00000027740.3:p.Asp768Glu). The codon is highly conserved in mammals (Table 4) and is an excellent functional candidate with a likely high impact on protein function (Polyphen score = 0.992). The third variant is an in-frame deletion (chrX.g.102369488-102369489insAAC; rs850984482) in exon 6 of the gene, which is in LD with the missense mutation. The deletion removes one polar amino acid, asparagine, in the conserved immunoglobulin-like domain (ENSCAFP00000027740.3:p.Asp376_Glu377insAsn) and is also a strong functional candidate.

**Table 4:** Conservation of *IGSF1* mutated codons (in bold) between mammals.

Species	IGSF1 Protein Size	% Identity with dog	Codon Deletion (codon 377)	Codon changing (codon 768)
Large Dog (SBW >41 kg [90 lb])	1338	-	TSIDD**-**ESFFLNNVTY	PFKWSEPS**D**PMELVIKE
Medium & Small Dog (SBW ≤41 kg)	1339	-	TSIDD**N**ESFFLNNVTY	PFKWSEPS**E**PMELVIKE
Human	1327	90.1	TSIDD**N**TSFFLNNVTY	PFKWSEPS**E**PLELVIKE
Mouse	1317	85.1	SSNTG**N**NSFFLKNVTY	PFKWSEPS**E**PLELVIKE
Cat	1341	95.2	ASTDD**D**ESFFLNNVTY	PFKWSEPS**E**PLELVIKE
Pig	1340	92.9	TSIDD**D**KSFFLNNVTY	PFKWSEPS**E**PLELVIKE
Cow	1327	90.5	TSVYD**D**TSFFLNNVTS	PFKWSEPS**E**PLELVIKE
Horse	1316	93	TSIDD**N**ESFFLNNVTY	PFKWSEPS**E**PLELVIKE
Megabat	986	74.6	TNIND**D**ESFFLNNVTY	PFKWSEPS**E**PLEVVIKX

The two potentially functional *IGSF1* mutations at locus 2 were considered further. To determine the ancestral allele for each, we used Sanger sequencing to ascertain genotypes from a set of wild canids, including 24 grey wolves from geographically diverse areas, two red wolves, and two coyotes. The two mutations (missense SNP at exon 12 and deletion at exon 6), while associated with large size in dogs, were never observed in the coyote, red wolves, or grey wolves, leading us to term these two large breed variants as “derived” alleles.

To determine the frequency of each candidate variant in domestic breeds, we used Sanger sequencing to analyze a large panel of 561 dogs encompassing 96 breeds (S6 Table). This panel included 10 additional large breeds and 36 more small/medium breeds. We observe both the exon 6 and 12 variations of *IGSF1* in the homozygous state in several large breeds of varying mass and skeletal size including the Bullmastiff, Great Dane, Great Pyrenees, Irish Wolfhound, Newfoundland, and Saint Bernard. Heterozygous genotypes were also identified in six additional large breeds: Black Russian Terrier, Dogo Canario, Greater Swiss Mountain Dog, Mastino Abruzzese and Tibetan Mastiff. As expected, based on this pattern, we never found the “large *IGSF1* allele” for either variant in the five unrelated Sanger sequenced Scottish Deerhounds, which are included in our definition of large breeds. We identified 17 medium and small breeds for which all sequenced dogs were homozygous for the derived allele at both *IGSF1* variants, and 21 medium and small breeds that were heterozygous, recapitulating the pattern observed above. Interestingly, among the medium and small breeds, we found muscled breeds such as Boston Terrier, Boxer, Bulldog, French Bulldog, Miniature Bull Terrier and Shar-pei. The remaining 36 small/medium breeds with SBW ranging from 2.7 to 39.5 kg (6.1 to 87 lb), and corresponding to 48.7% of the medium/small breeds, were homozygous for the ancestral alleles for both variants (S6 Table). The “large alleles” were found in 95% of large breeds, and the genotypes appeared fixed (homozygous for the “large alleles”) in 76.2% of large breeds. By comparison, 51.4% of medium/small dogs carry what we considered to be “large alleles” (homozygous in 44.7%). This argues that while *IGSF1* likely plays a role in modulating weight variation in modern breeds, it is also, and more precisely, a contributor to the muscled phenotype in breeds spanning a range of body sizes (Fig 4).

## Discussion

In this study, we identified two loci on the X chromosome associated with SBW in domestic dog breeds, using a panel of 855 dogs selected to represent the full range of canine body size, which we genotyped on the Illumina Canine HD SNP array. We showed that two large haplotypes at two loci were shared by the majority (>90%) of large breeds with SBW >41 kg (90 lb), for which derived alleles (not present in wolves) have been identified. Fine mapping using whole genome sequencing data from 163 dogs revealed candidate variants in *IRS4* and *IGSF1* that are strongly associated with large breeds. Interestingly, we also identified a phenotype of bulky or stocky build, which is also referred to as “heavily muscled,” for which a third candidate gene, *ACSL4*, and variant were associated. The bulky haplotype was found post hoc and not detected by either our GWAS or any previously published GWAS, because no SNP on the canine HD SNP array is in LD with the variants. These particular allelic distributions in the canine population highlight the strong impact of X chromosome genes in determining the weight and muscling of modern dog breeds.

Our previous studies identified alleles in the *GHR*, *HMGA2*, *SMAD2*, and *STC2* genes as major contributors to SBW [47]. When we included genotyping data from *IGF1* and *IGF1R*, which we had identified previously as body size genes in dogs [41,42], we showed that these six genes explain about 60% of body size variance in small/medium breeds, but <5% of variance in large breeds. This highlights a now recurring theme in dog genetics that a small number of genes of large effect control many complex phenotypes, as opposed to many genes of small effect as is observed often in humans.

We used two different approaches to identify variants associated with large body size. SNP chip data were used to identify large regions of LD. However, this strategy does not detect rare variants that are not in LD. WGS provides a complementary tool for these types of analyses. Indeed, this allows detection of rare mutations that would otherwise go unnoticed. In this study, the combination of dense SNP chip data (Illumina 170k) and WGS highlighted rare variants, such as the *ACSL4* mutation, which are specific to a subset of large breeds, a result not found with SNP chip data alone. This approach allowed us to define a new and very specific phenotype, the heavy muscling trait, which had not been previously described in dogs at a genetic level.

We found first that *IRS4* is strongly associated with large body size in dogs. The gene encodes a cytoplasmic protein that contains several potential tyrosine and serine/threonine phosphorylation sites. IRS4 interacts with multiple growth factor receptors such as IGF1R, enhancing IGF1-stimulated cell growth [49]. This gene is highly expressed in the hypothalamus which itself plays a primary role in regulation of body weight [56]. It is also estrogen-regulated [62], which may explain, in part, the established link between estrogen and body fat distribution [63]. Moreover, a double “knock-out” mouse model (*bIrs2-/-*.*Irs4-/y*) developed severe obesity suggesting that IRS4 synergizes and complements IRS2 [64]. In humans, six SNPs in *IRS4* have been identified that are associated with obesity, albeit in a cohort of patients with schizophrenia [56]. In our study, we identified three genomic variations in *IRS4*. Neither the codon deletion nor one codon insertion in the exonic region of *IRS4* appeared to be associated with disruptions in protein function. However, in large bulky/muscled breeds, we also detected an associated 56 kb deletion located 150 kb upstream of the start codon of *IRS4*. This deletion contained several repeated elements, and may contain regulatory elements that affect the expression profile of *IRS4* [65,66].

While no correlation was found between height and *IRS4* in the human study [56], in our canine study we observe a strong correlation between *IRS4* and SBW that extends to include standard breed height (SBH) (S4 Fig). SBH is the height range assigned by the AKC for a given breed. However, the addition of the SBH as co-variate in primary GWAS results in the loss of the locus 1 signal. Interestingly, the reverse analysis confirms the strong association between SBW and both loci. Indeed, the addition of SBW as a covariate for the SBH GWAS results in the loss of both signals on the X chromosome (S4 Fig). Overall this suggests that while both *IRS4* and *IGSF1*, the latter of which is the second candidate gene on X chromosome, are associated with variation in breed size, *IRS4* is necessary, but not sufficient, for increasing size.

We also showed that the *IGSF1* gene, positioned at a second locus on the X chromosome, is strongly associated with large dog breeds. This gene encodes a plasma membrane glycoprotein and is involved in the thyroid hormone pathway [50]. In large dog breeds, we identified two mutations, one single codon deletion and one missense mutation, both of which are located in a highly conserved immunoglobulin-like domain of IGSF1 protein. In humans, mutations in the same IGSF1 protein domain are associated with the X-linked IGSF1 deficiency syndrome [50–52,67–69]. Some patients show growth hormone (GH) deficiency during childhood, and 67% of male children are reportedly overweight while 21% are obese (Review in [70]). The general observation is supported by the fact that Igsf1-deficient male mice show diminished pituitary and serum thyroid-stimulating hormone (TSH) concentrations, reduced pituitary thyrotropin-releasing hormone (TRH) receptor expression, and increased body mass [50]. Measuring these hormone levels in dogs, while difficult, may confirm the parallels between dogs and mice.

We also detected a strong association between *IGSF1* and SBH (S4 Fig). Human studies used body mass index (BMI) as a measure of obesity given a particular height. To date, 97 loci are associated with human BMI [71]. It could be interesting to develop the same body mass index measure for dogs to better understand the results regarding *IGSF1*, *IRS4*, *ACSL4*, *IGF1*, *IGF1R*, *HMAGA2*, *GHR*, *SMAD2*, and *STC2*. This approach could explain why our study revealed that 50% of small/medium breed dogs have the “large alleles,” mainly found in muscled breeds such as Boston Terrier or French Bulldog (Fig 4). Interestingly, the *IGSF1* locus also appears to be under selection in GWAS studies for other morphologic traits, such as brachycephalic (*e*.*g*. bulldog, pug) versus dolichocephalic (*e*.*g*. afghan hound, collie) skull shape [22,72] (Fig 4). In humans, patients with microduplication of the *IGSF1* locus present syndromic facial appearance [73]. The varying phenotypes associated with *IGSF1* illustrate the intermingling of genes and phenotypes regarding skeletal formation.

In addition to breed standard weight and heights, this study revealed a genetic association with a well-defined phenotype of bulkiness, due to heavy muscling and fat, which we found to be strongly associated with a highly conserved single nucleotide in the 5’ UTR in canine *ACSL4* at locus 1 (S3 Fig). *ACSL4* belongs to the long-chain acyl-CoA synthetase (ACSL) family and five genes have been identified in mammals (*ACSL1*, *3*, *4*, *5*, and *6*) [74,75]. *ACSL4* binds specifically to longer chain polyunsaturated fatty acids. While *ACSL4* plays a role in many cellular processes [76–80], increased *ACSL4* expression in the liver likely promotes fatty acid uptake [53]. The relationship between the gene and body shape in dogs fits well with this observation. We did not observe the same relationship between *ACSL4* and stocky dogs from small breeds, suggesting that the genetic variant found in large dogs is not relevant in the absence of genes that increase body size.

In the pig, mutations in *ACSL4* are associated with a phenotype termed “back fat thickness (BFT)”. There are 75 common breeds of pigs (http://www.thepigsite.com/) and large variation in adiposity between breeds has been described [58]. Pig breeds with considerable back fat are used to study human obesity as well as obesity-related diseases, such as metabolic syndrome [81]. Four QTLs on the porcine X chromosome were associated with the BFT, muscle mass, and intramuscular fat content [57,59]. Post-mortem studies reveal that polymorphisms surrounding the *ACSL4* gene are associated with BFT and muscle-associated traits in a pig breed-specific manner [57] as was observed in dog breeds within our study. Specifically, the canine variant (chrX.g.82919525C>T) was observed only in bulky dogs including, for instance, the Bullmastiff, Greater Swiss Mountain Dog, Newfoundland and Saint Bernard. All of these breeds are well-muscled breeds compared to the leaner Great Dane, Borzoi which, for example, lack the variant. The absence of the derived *ACSL4* allele in more than 97% of breeds which meet the definition of medium/small, as well as in giant thin breeds led us to define the “bulky phenotype” in dogs characterized by the traits of heavy muscling and back fat thickness which, together, are observed in 54% of the large breeds. We also notice an interesting correlation between the presence of the derived allele in some “large breeds” and their historic geographic distribution (S5 Fig). The “bulky allele” seems to have appeared in England-France (Dogue de Bordeaux, English and Bullmastiff), become fixed in these breeds, and then spread through Europe (Bernese Mountain Dog, Leonberg, Kuvasz). Mediterranean and Eurasian breeds (Cane Corso, Neapolitan Mastiff, Anatolian Shepherd) do not have this allele, likely reflecting the recent geographic spread of the allele in Europe. Finally, additional studies in pigs describe two mutations in the *IRS4* gene, perhaps suggesting a second role for *IRS4* as a contributor to BFT as well as general body size [57,82].

In this study, we utilized WGS and GWAS to identify genes highly associated with large body size in dogs. Modern dogs display a range of traits that have been easily mapped by taking advantage of the long LD observed in many breeds. That same LD makes it problematic to go from associated marker to gene. The availability of WGS represents a major advance for tackling this issue and, in this case, allowed us to disentangle the genetics of a complex trait on a relatively homogenous chromosome. While a large number of genes of small effect seem to control body size in humans, in dogs a surprisingly small number of genes of large effect explain the range in size observed across breeds. As dogs at the extremes of the body size continuum are studied, it will be interesting to note if genes previously identified from human studies are identified, or if an entirely new repertoire of genes are found which contribute to gigantism or miniaturization of breeds. Studies in domestic dogs, therefore, provide a mechanism for understanding the genetics that underlies traits of interest in both human and domesticated animals.

## Methods

### Sample collection and DNA extraction

Whole blood samples were collected into EDTA or ACD anticoagulant from AKC-registered dogs. Genomic DNA was extracted using a standard phenol-chloroform extraction protocol [83]. All procedures were reviewed and approved by the NHGRI Animal Care and Use Committee at the National Institutes of Health.

### Phenotype assignment

Standard breed weights and height were obtained from several sources: weights previously listed in Rimbault et al. [47] were used, although they were updated if weights specified by the AKC [84] were different. If the AKC did not specify SBW and SBH, we used data from *Atlas of Dog Breeds of the World* [16]. SBW and SBH (male + female average) were applied to all samples from the same breed and the values used in this study are listed in S1 and S2 Tables. Analyses by sex did not change the results, thus we retained the genotypes as a single dataset.

### SNP genotyping

Genotyping was performed using the Illumina 170K Canine HD SNP array containing approximately 170,000 SNPs distributed across the 38 canine autosomes and the X chromosome. Genotypes were called using Illumina Genome Studio software. In total, 855 dogs, 418 males and 437 females, were genotyped [21]. Dogs belong to 88 different breeds. Eighty-two breeds with nine to 11 dogs were genotyped and six large dog breeds with four to six dogs genotyped. All samples had a call rate greater than 93% (range: 93.57–99.98, average: 99.84). SNPs with a minor allele frequency <1% or the presence of >5% missing genotypes were pruned, resulting in a final dataset of 150,895 SNPs that were used for the subsequent GWAS. The GWAS was conducted using the software GEMMA v0.94.1 (Genome-wide Efficient Mixed-Model Association) [54,55] as a linear mixed-model software using a centered kinship matrix. Pedigrees of dogs used in the study were verified to avoid inclusion of close relatives, *i*.*e*. none shared a common grandparent. In the two regions of interest, pairwise r^2^ values were calculated using Plink v1.07 [85].

### Whole Genome Sequencing

Fine mapping at both loci used data from 157 individuals who had undergone WGS and for which the data were published or available online from the Sequence Read Archive (http://www.ncbi.nlm.nih.gov/sra). Six new WGS recently produced by the NIH Intramural Sequencing Center (NISC) were also included. The latter were produced using the Illumina TruSeq DNAPCR-Free Protocol (Cat.FC-121-3001). Reads were aligned to the CanFam 3.1 reference genome (http://genome.ucsc.edu/cgi-bin/hgGateway?db=canFam3) using BWA 0.7.13 MEM [86] and sorted using SAMtools 1.3.1 [87]. PCR duplicates were marked as secondary reads using PicardTools 2.2.4 (http://github.com/broadinstitute/picard) for those libraries that were not PCR-free. GATK 3.5 [88,89] was used to perform local realignment around putative indels events using 714,278 variants published in [90] as the training set. A total of 172,254 Illumina Canine HD Chip positions and 2,738,537 dbSNP v131 variants were utilized for base recalibration with GATK 3.5. SNV were called per-individual in gVCF mode of HaplotypeCaller [91], with subsequent joint-calling across all individuals. Variant quality score recalibration was conducted with GATK best practices and default parameters for SNV and indels separately as follows. Indel recalibration: 714,278 variants as truth and training sets with a prior of six [90]. SNV recalibration: 172,254 Illumina Canine HD Chip variants (known, training, true, prior = 12); 2,738,537 dbSNP v131 variants (known, true, prior = 8); 3,627,539 published variants from [92] (known, training, prior = 6). We only used genomes with a sequencing depth >10X and retained only variants with a minimum of two alleles and a minor allele frequency >5%. For locus 1, 6,809 variants met the QC criteria, while 1,997 met the criteria for locus 2. These variants were analyzed using GEMMA v0.94.1 as a linear mixed-model software [54,55]. A centered kinship matrix was estimated extracting SNPs from the 163 WGS data using the positions of 147,740 SNPs of the Illumina Canine HD SNP array. DELLY and CNVnator were used to analyze structural variants, including indels, inversions and duplications that were >100 bp in length [92,93].

### Sanger sequencing

To confirm the distribution of “large alleles” in the *IRS4* and *IGSF1* genes, we genotyped a panel of 512 dogs of 93 breeds and 24 wolves (S6 Table). Primer pairs were designed to target regions that included the variants of interest, and two pairs were specifically designed to reveal the absence/presence of the deletion (S7 Table). Targeted regions were assayed using polymerase chain reaction (PCR) with AmpliTaq Gold. PCR products were purified by ExoSap-It reaction (Affymetrix), and then Sanger sequenced using BigDye Terminator v3.1 (Applied Biosystems). Products from sequencing reactions were run on ABI 3730 DNA analyzer. Sequence traces were analyzed using Phred/Phrap/Consed package [94–96]. The absence/presence of the deletion was detected after migration of the PCR products on a 1% agarose gel followed by staining with ethidium bromide. To analyze the *ACSL4* variant, we sequenced a larger set of 985 unrelated dogs and wild canids, including 24 geographically diverse gray wolves from North America, Europe, and Asia, two coyotes and two red wolves (S5 Table). Three hundred and fifteen of these dogs were included in the dataset used for the initial GWAS.

### Conservation between species

To estimate the conservation of mutated codons/nucleotides between mammals, we used both protein and gene sequences from *IRS4*, *IGSF1* and *ACSL4* which were available on Ensembl [97]. We selected proteins for dog, human, mouse, cat, pig, horse, cow, and megabat and we used SIM [98] and LALNVIEW [99] to align sequences.

## Supporting information

S1 Fig Summary of genetic investigations of X chromosome locus 1 provided by dog model, SNP chip and WGS data. (TIF)

S2 Fig Summary of genetic investigations of X chromosome locus 2 provided by dog model, SNP chip and WGS data. (TIF)

S3 Fig UCSC screenshot of the conservation between mammals of the 5’UTR variant in *ACSL4* (red square). (TIF)

S4 Fig Standard breed weight and standard breed height GWAS results. Manhattan plots using Standard Breed Weight without (A) and with Standard Breed Height as a covariate (B). Manhattan plots using Standard Breed Height without (C) and with Standard Breed Weight as a covariate (D).
(PDF)

S5 Fig Historical distributions of the large breeds and the “bulky allele”. (TIF)

S1 Table List of the 855 dogs genotypes on the Illumina Canine HD SNP array. (XLSX)

S2 Table List of the 163 dogs that underwent WGS for this analysis of the X chromosome. (XLSX)

S3 Table Variants identified at locus 1 by fine-mapping using WGS data from 163 dogs. (XLSX)

S4 Table Variants identified at locus 2 by fine-mapping using WGS data from 163 dogs. (XLSX)

S5 Table Lists of dogs used for Sanger sequencing of the 5’UTR mutation in *ACSL4* and the intronic *AMMECR1* variants. (XLSX)

S6 Table Lists of the dogs used for Sanger sequencing for the candidate mutations in *IGSF1*. (XLSX)

S7 Table Primers used for PCR and Sanger sequencing. (XLSX)
