# Supplementary figures and images for: Analysis of large versus small dogs reveals three genes on the canine X chromosome associated with body weight, muscling and back fat thickness
Source: PLoS Genet. 2017 Mar 3;13(3):e1006661. doi: 10.1371/journal.pgen.1006661 (PMC5357063; doi:10.1371/journal.pgen.1006661)

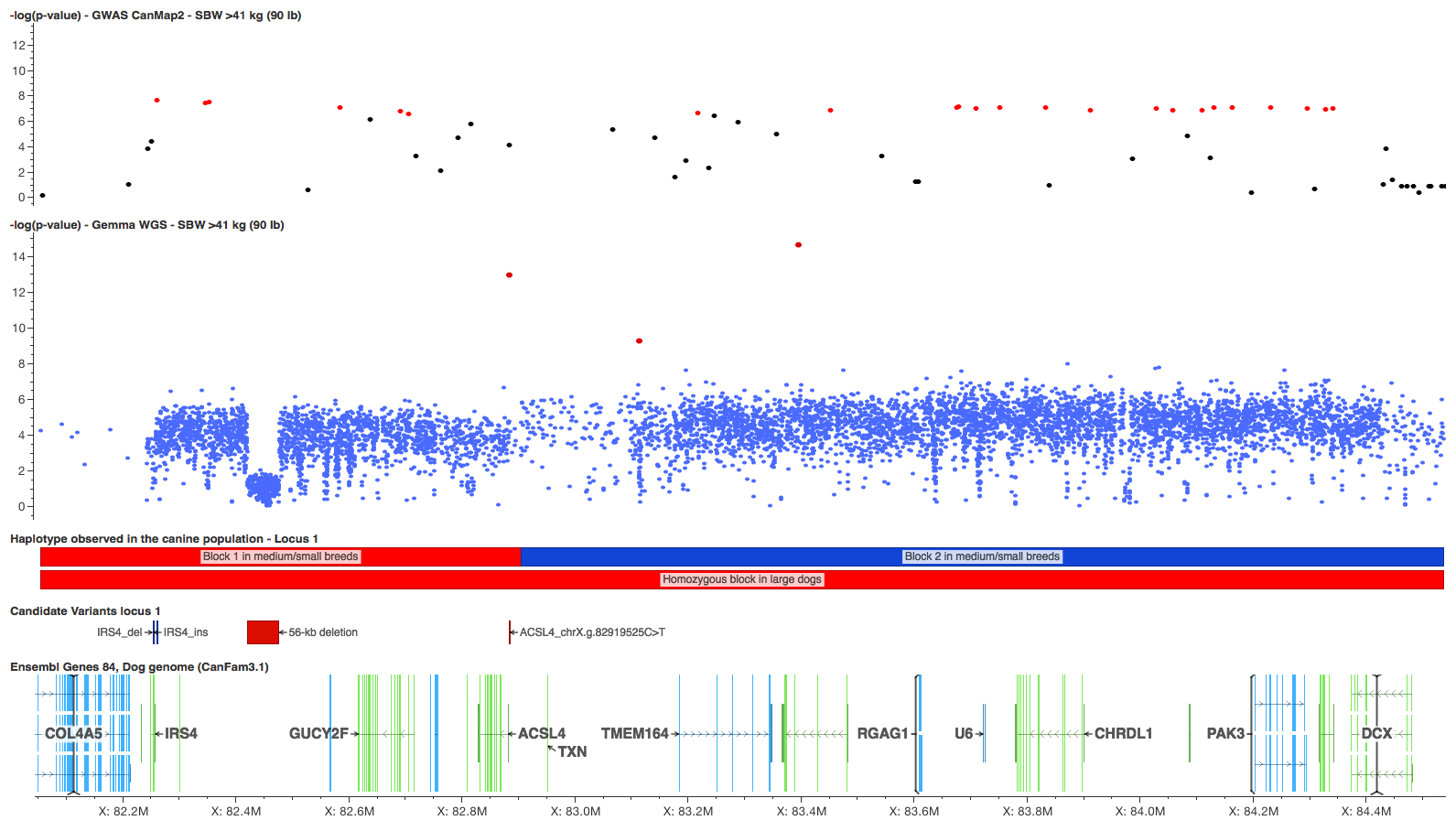

Supplement: S1 Fig — (TIF) [file pgen.1006661.s001.tif]

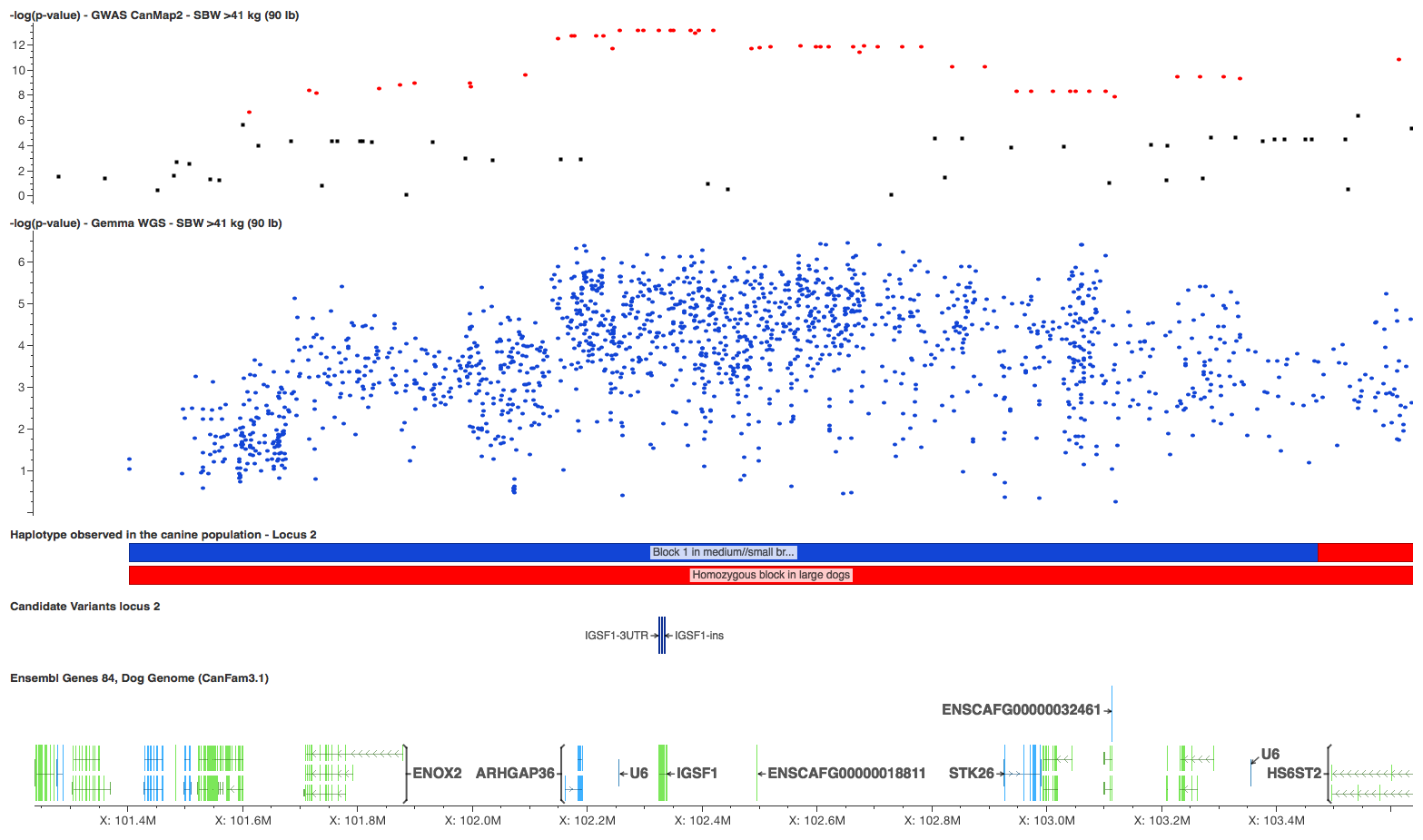

Supplement: S2 Fig — (TIF) [file pgen.1006661.s002.tif]

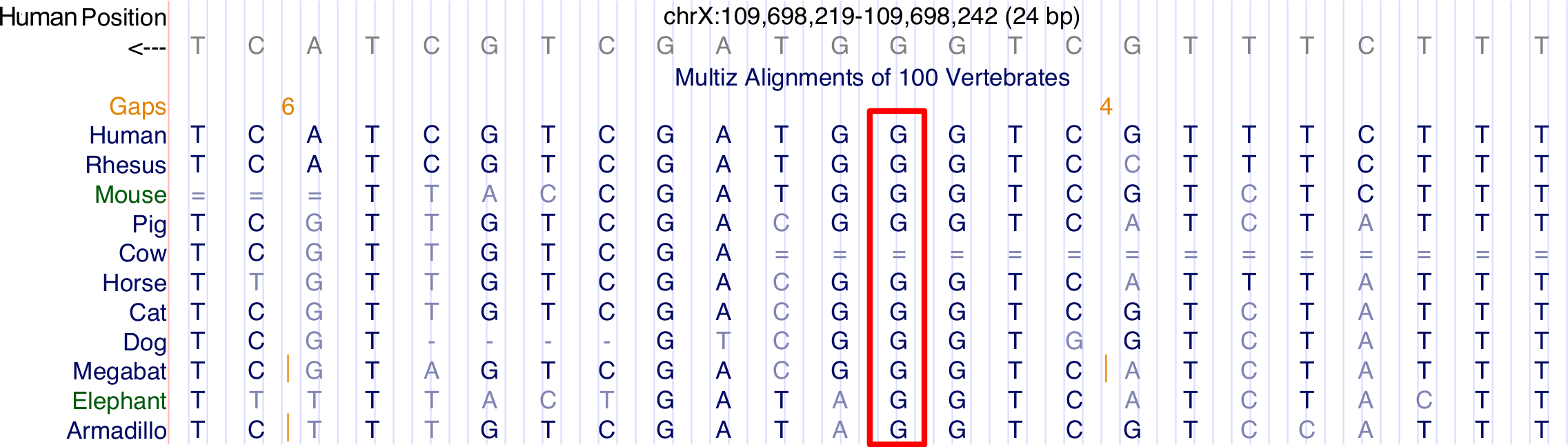

Supplement: S3 Fig — (TIF) [file pgen.1006661.s003.tif]

A

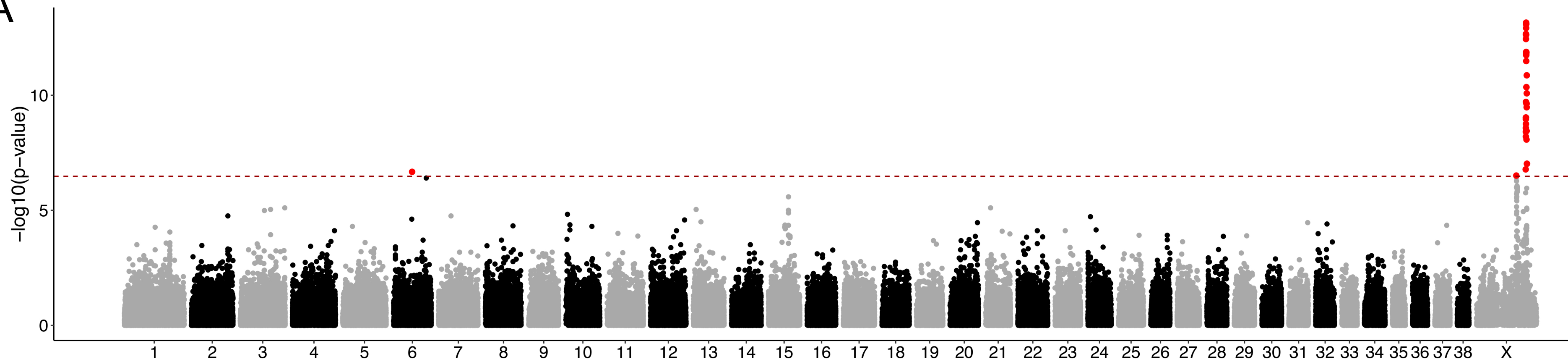

B

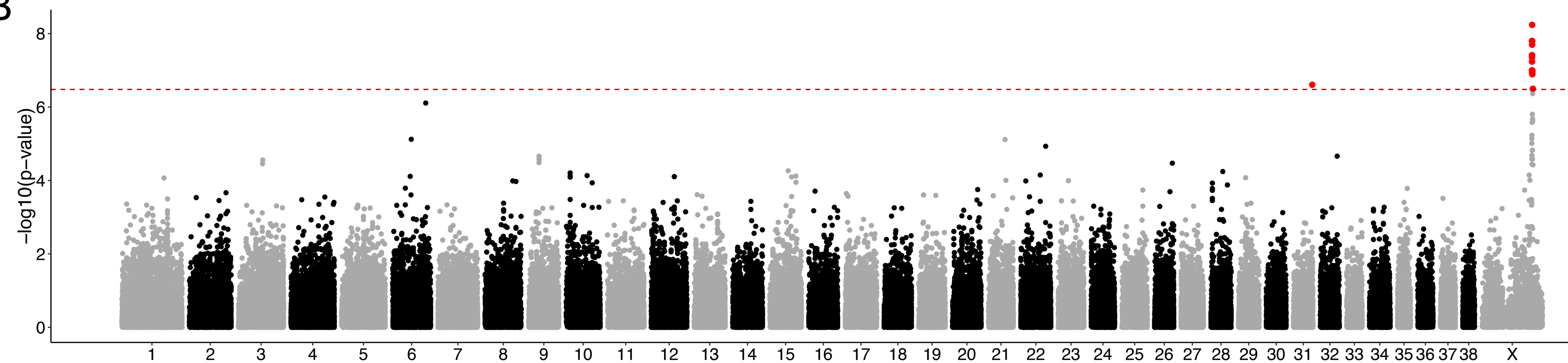

C

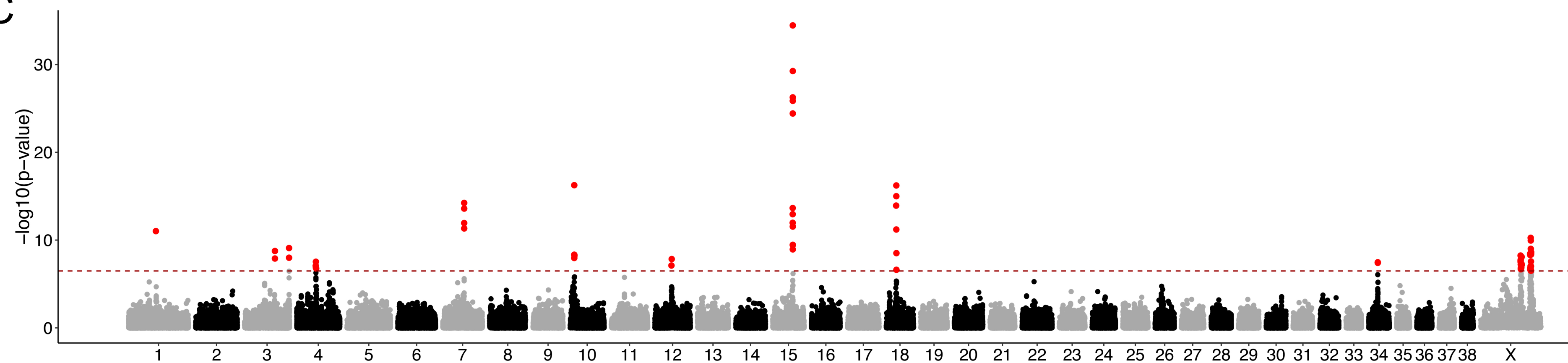

D

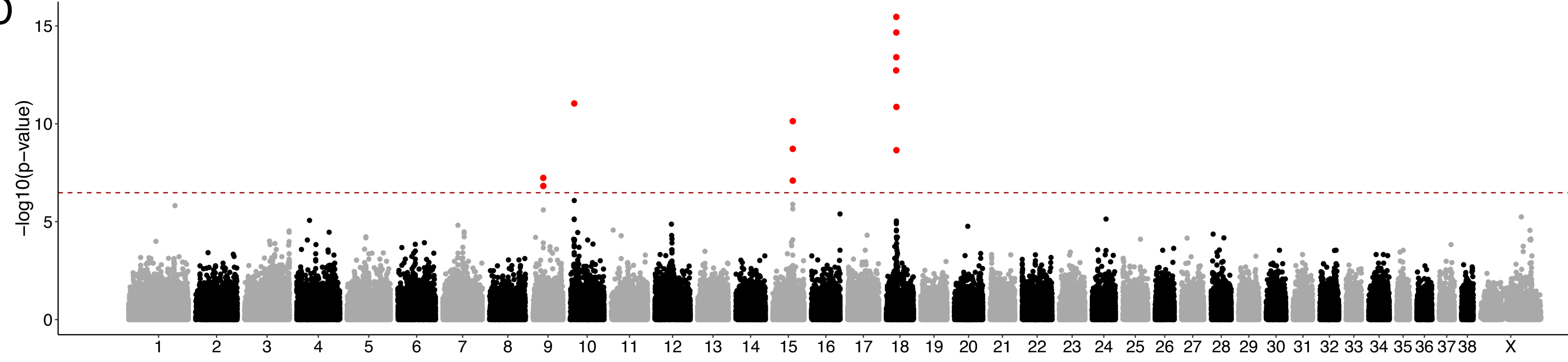

Supplement: S4 Fig — Manhattan plots using Standard Breed Weight without (A) and with Standard Breed Height as a covariate (B). Manhattan plots using Standard Breed Height without (C) and with Standard Breed Weight as a covariate (D). (PDF) [file pgen.1006661.s004.pdf]

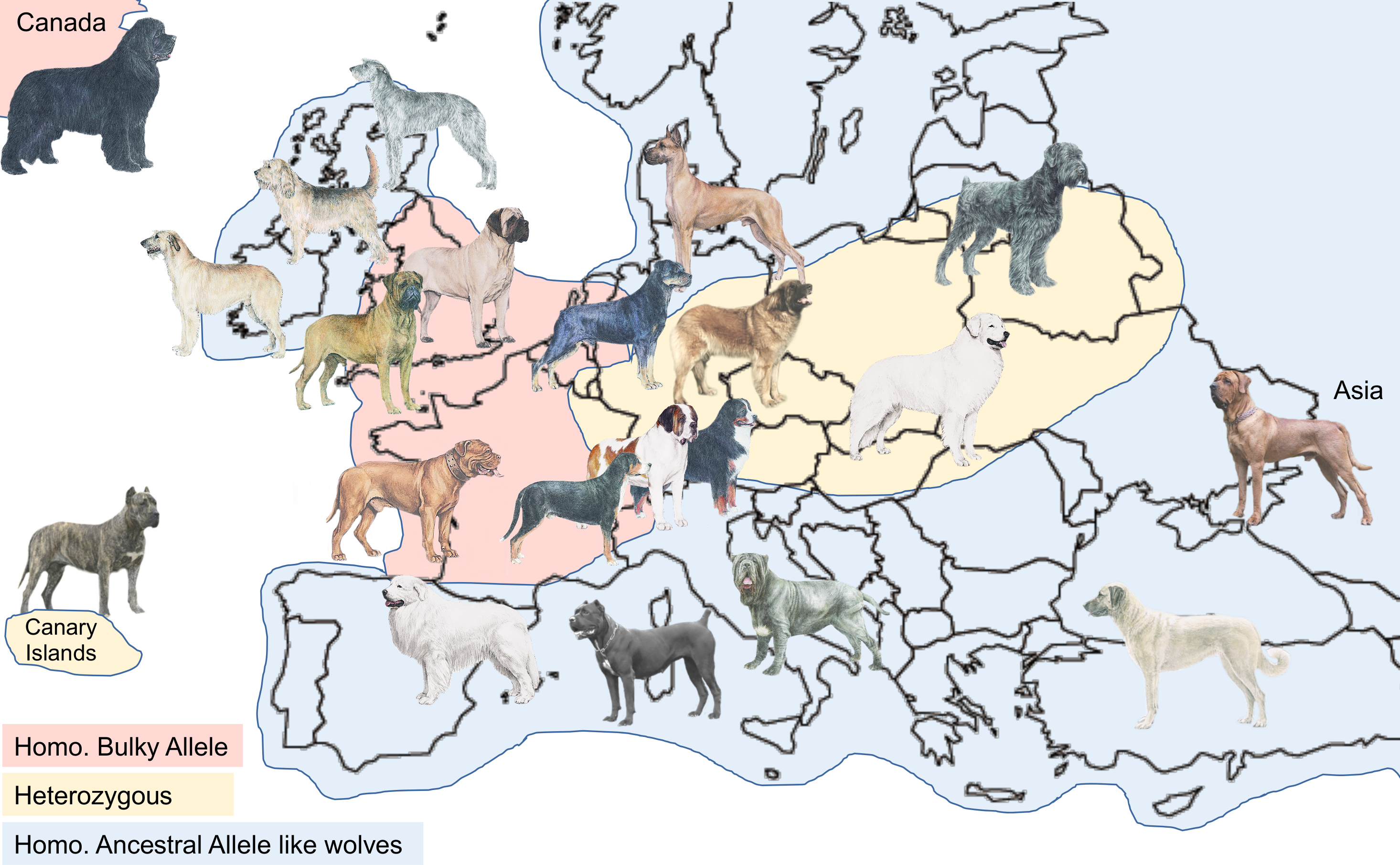

Supplement: S5 Fig — (TIF) [file pgen.1006661.s005.tif]
